# A Bidirectional Brain-Machine Interface Featuring a Neuromorphic Hardware Decoder

**DOI:** 10.3389/fnins.2016.00563

**Published:** 2016-12-09

**Authors:** Fabio Boi, Timoleon Moraitis, Vito De Feo, Francesco Diotalevi, Chiara Bartolozzi, Giacomo Indiveri, Alessandro Vato

**Affiliations:** ^1^Neural Computation Laboratory, Istituto Italiano di TecnologiaRovereto, Italy; ^2^Institute of Neuroinformatics, University of Zurich and ETH ZurichZurich, Switzerland; ^3^Robotics, Brain and Cognitive Sciences, Istituto Italiano di TecnologiaGenova, Italy; ^4^iCub Facility, Istituto Italiano di TecnologiaGenova, Italy

**Keywords:** bidirectional BMI, neuromorphic decoder, on-line learning, modular system, spiking neural network

## Abstract

Bidirectional brain-machine interfaces (BMIs) establish a two-way direct communication link between the brain and the external world. A decoder translates recorded neural activity into motor commands and an encoder delivers sensory information collected from the environment directly to the brain creating a closed-loop system. These two modules are typically integrated in bulky external devices. However, the clinical support of patients with severe motor and sensory deficits requires compact, low-power, and fully implantable systems that can decode neural signals to control external devices. As a first step toward this goal, we developed a modular bidirectional BMI setup that uses a compact neuromorphic processor as a decoder. On this chip we implemented a network of spiking neurons built using its ultra-low-power mixed-signal analog/digital circuits. On-chip on-line spike-timing-dependent plasticity synapse circuits enabled the network to learn to decode neural signals recorded from the brain into motor outputs controlling the movements of an external device. The modularity of the BMI allowed us to tune the individual components of the setup without modifying the whole system. In this paper, we present the features of this modular BMI and describe how we configured the network of spiking neuron circuits to implement the decoder and to coordinate it with the encoder in an experimental BMI paradigm that connects bidirectionally the brain of an anesthetized rat with an external object. We show that the chip learned the decoding task correctly, allowing the interfaced brain to control the object's trajectories robustly. Based on our demonstration, we propose that neuromorphic technology is mature enough for the development of BMI modules that are sufficiently low-power and compact, while being highly computationally powerful and adaptive.

## 1. Introduction

The possibility of controlling a prosthetic device through a direct interface with the central nervous system represents a promising solution for restoring sensory-motor functionalities in patients with limb amputations or peripheral and neurological deficits due to spinal cord injury, amyotrophic lateral sclerosis, or stroke. In the last two decades, a fast-growing worldwide scientific community has developed several brain-machine or brain-computer interfaces (respectively, BMIs or BCIs) toward the clinical application of these devices. Such interfaces offer also a powerful tool for exploring the sensory-motor mechanisms of control, adaptation, and learning that are employed by the central nervous system. This research has been assisted both by progress in our understanding of the underlying neural processes that take place in the brain, and by technological advances that have dramatically improved the quality of the signals recorded from the brain and the possibility of managing and processing large amount of data in real-time (Wolpaw et al., 2000; Lebedev and Nicolelis, 2006; Wander and Rao, 2014). Encouraging results have been recently obtained in controlling a robotic arm by using motor neural activity in tetraplegic patients (Hochberg et al., 2012) and by restoring cortical control of movement in humans with quadriplegia (Bouton et al., 2016) but these setups still have limitations that prevent their clinical use on a large scale (Baranauskas, 2014).

The development of a BMI system aiming for large clinical application requires crucial improvements of the hardware and software components. The hardware components need to be (a) fully implantable for long term use and therefore miniaturizable; (b) able to reliably process neural signals with a limited power budget; (c) powerful enough to implement non-trivial computational tasks involved in a BMI system. Additionally, the decoding algorithms need to be (d) sufficiently flexible to be implemented with different types of hardware components, and (e) able to dynamically adapt to changes in the neural activity due to the interaction with the artificial device (Dangi et al., 2011; Orsborn et al., 2014).

Neuromorphic devices comprise compact, energy-efficient, and adaptive circuits that have been demonstrated to be optimal for tasks that involve learning from real-world observations in an on-line fashion (Chicca et al., 2014). They achieve this by employing silicon emulations of biological neurons and synapses that can be physically configured to implement algorithms inspired by the asynchronous massively parallel computations performed in biological neural networks. Additionally, input to and output from neuromorphic chips is provided with asynchronous digital pulses that encode information in their analog timing, similarly to action potentials of biological neurons. Because of these features, neuromorphic processing chips are very promising candidates for implementing reliable and energy-efficient decoding of neural activity, that could ultimately be evolved to be portable, implantable, and directly interfaced with neural tissue.

For this reason we directed our efforts toward the development of a fully implantable BMI by prototyping a neuromorphic processor chip (Qiao et al., 2015) integrated in a bidirectional brain-machine interface, trained to decode neural signals recorded on-line, and to provide suitable outputs useful for controlling actuators and end effectors. In order to assess the performance of this system, we took the following steps: first we developed suitable spike-based decoding methods that could be implemented by the neuromorphic processor chip, then we configured the chip to implement these methods in real-time and adapted the bidirectional BMI designed and tested in our lab (Vato et al., 2012) to include in the processing chain this neuromorphic component. Finally, we tested this neuromorphic bidirectional BMI in a closed-loop real-time experimental setup that involved the control of the motion of an external device by the decoded neural signals recorded from the brain of an anesthetized rat. Here, we describe in detail the properties of the neuromorphic processor, and the network of spiking neurons that was implemented by the chip to carry out the decoding task. We present the main hardware and software modules that we developed to interface the chip with the other components of the BMI, and describe the experimental paradigm that we used to test the system.

Our approach differs from those of currently-developed BMIs, which are *ad hoc* ensembles of hardware and software elements designed to perform specific tasks, and which are difficult to replicate, generalize, or modify for use in other tasks or different environments (Leuthardt et al., 2006). As these are limitations that hinder collaborations between laboratories we chose to emphasize a modular approach in designing our BMI by developing a system that is compatible with a wide range of different hardware and software standards, and which is composed of a main control core module and multiple possible recording, stimulating, decoding, and encoding modules. We argue that the combination of this modular bidirectional BMI setup with the use of neuromorphic hardware modules can give a crucial contribution to the development of the next generation of brain-machine interfaces for large-scale clinical applications.

## 2. Materials and methods

We begin by describing the general scheme of this novel bidirectional BMI in Section 2.1 and the experimental procedure used to test the performance of the neuromorphic decoder in Section 2.2. In Section 2.3, we describe in details the main modules comprising the system and finally we present the hardware and the software implementation of the neuromorphic chip, respectively, in Sections 2.4 and 2.5.

### 2.1. General scheme of the modular bidirectional BMI

**Figure 1:**
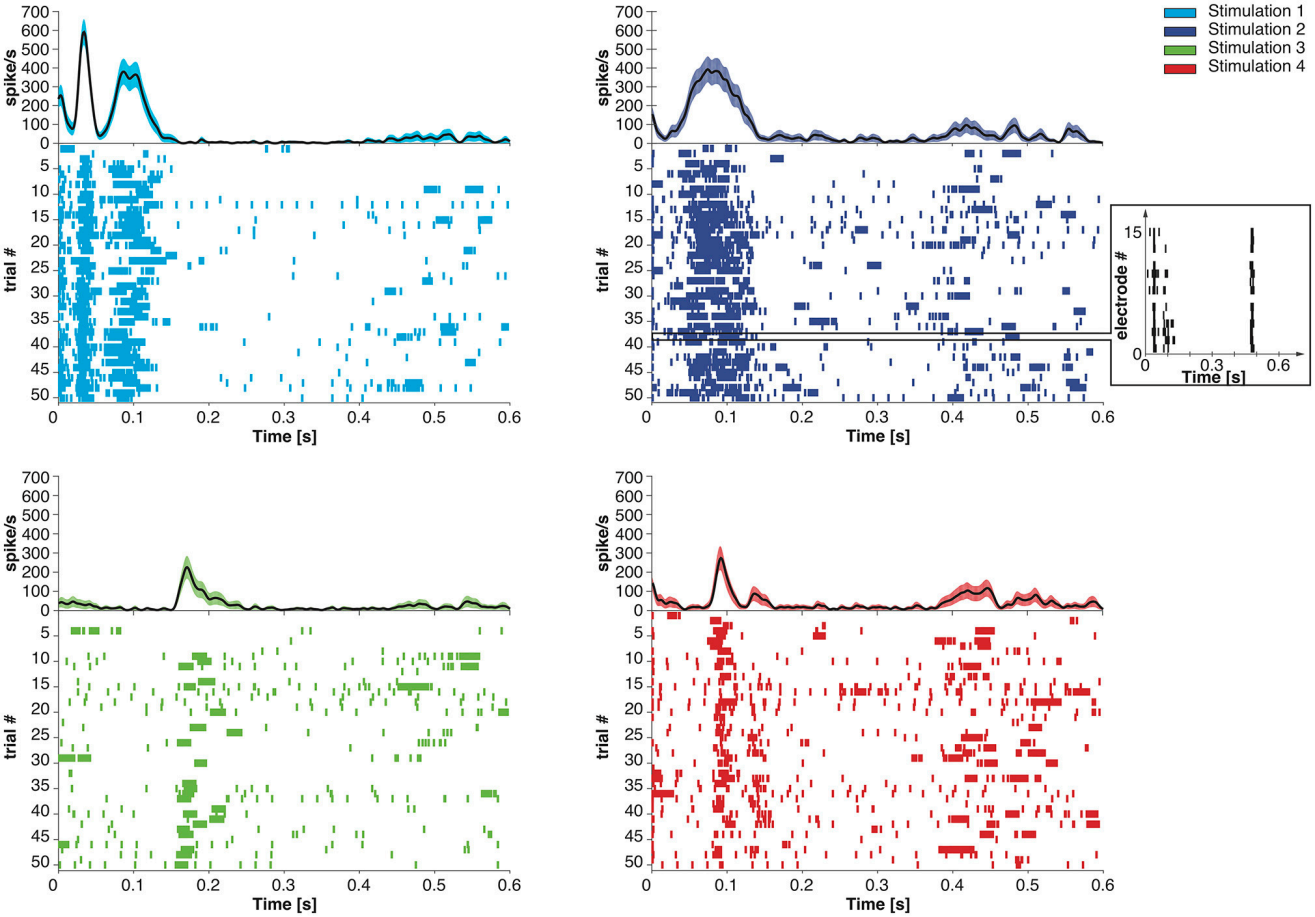
**Post-stimulus time course of the time-dependent firing rate (mean ± SEM across trials) and raster plot of the recorded neural activity evoked by four different stimulation patterns**. Each short vertical line in the raster plots represents the occurrence of at least one spike recorded from all the electrodes on the recording array in a 1 ms time bin. In the inset, we report the neural activity recorded from each electrode of the microwire array during a single trial.

We extended the Dynamic Neural Interface described in Szymanski et al. (2011) and Vato et al. (2012, 2014) with the inclusion of a neuromorphic decoder module. This system uses the neural signals collected from a rat's brain to control the movement of an external object by means of a sensory and motor interface. In designing it we took inspiration from earlier studies in frogs (Bizzi et al., 1991), rats (Tresch and Bizzi, 1999), and cats (Lemay and Grill, 2004) by emulating the functioning of the spinal cord that combines sensory information with brain instructions and organizes the movement of the limbs along dynamically stable trajectories. We set up a decoding and an encoding interface which generate a dynamic control policy in the form of a force field and robustly drive the movement of the controlled object. The neural signals are recorded from the motor cortex of the anesthetized rat by means of a recording multielectrode array. These signals are transformed by the decoder into a force vector to be applied to a device that can control the motion of the object. After receiving this external input, the device moves the object, according to its dynamics, for a predefined amount of time. An encoder maps each position of the object in the workspace to a pattern of intracortical microstimulation (ICMS) delivered to the somatosensory cortex of the rat. This is achieved by means of a stimulating multielectrode array which provides the brain with information about the position of the controlled object. A calibration procedure of the interface establishes a control policy based on an approximation of a radial force field with the aim of driving the controlled object toward a target location defined by the central equilibrium point of the field. In the implementation described here we use four different patterns of intracortical stimulation and, consequently, the workspace is divided into four different contiguous sensory regions. The four stimulation patterns differ from each other only in the combination of the electrodes chosen to deliver the stimulation. Each stimulation pattern consists of a train of 10 biphasic pulses (100 μA, 100 μs/phase, cathodic first) delivered at 333 Hz (Butovas and Schwarz, 2007; Semprini et al., 2012). After each stimulation, the decoder considers the first 256 ms of the evoked motor neural signal to produce the driving force for the external device. In Figure 1, we report the post-stimulus time course of the time-dependent firing rate (mean ± sem over 50 trials) of the evoked neural activity recorded from all the electrodes of the array. The raster plots represent the time occurrences of at least one spike recorded from all the electrodes of the multielectrode array.

The calibration force corresponding to each region was defined by a vector pointing from the region's centroid to the target (colored thick arrows depicted in **Figure 8**). The task of the decoder consists in extracting from each evoked neural response a resulting force, calculated as a weighted sum of the four calibration forces defining the force field. In particular, the decoder needs to extract the four coefficients corresponding to the contribution of each of the four calibration forces to the decoded force.

### 2.2. Experimental procedure

Neural data were collected from male Long-Evans rats (300–400 g) anesthetized for the entire duration of the experimental sessions by means of Xylazine (5 mg/kg) and a mixture of Tiletamine and Zolazepam (30 mg/kg). Two craniotomies were performed above the somatosensory (S1) and the motor (M1) cortex representing the whiskers on the same hemisphere. The stimulation microwire array (Tucker Davis Technologies—TDT) was lowered perpendicular to the somatosensory cortex 300–500 μm under the surface (AP −3.5 mm, LM +4 mm with respect to the most posterior medial electrode of the array). The recording array was placed at depth 900–1100 μm below the pia (AP −1.5 mm, LM +0.5 mm with respect to the most posterior medial electrode of the array) using a hydraulic microdrive. These locations have been chosen for the presence of several cortico-cortical connections between the two regions(Mao et al., 2011). Both arrays are composed of 16 microelecrodes (2 rows of 8 electrodes, 50 μm diameter) each one separated from the neighboring ones by 250 and 375 μm along and across the rows, respectively. All the experiments have been performed in accordance with DL116/92 of the Italian legal code and approved by the institutional review board of the University of Ferrara and by the Italian Ministry of Health (73/2008-B).

### 2.3. Main modules of the BMI system

The modular bidirectional BMI was designed around a core unit named Managing Unit (MU) that can be connected to satellite modules, each dedicated to specific tasks as decoding the neural signal, controlling the movement of an external device, and encoding the information collected from the external environment to provide sensory feedback. The MU does not require any information about the specific implementation of each module, which can be a software running on general purpose processing units, a dedicated programmable hardware such as Field Programmable Gate Arrays (FPGA) or a neuromorphic chip. This modularity ensures a fast and flexible prototyping phase required during research and development, whereby different software modules can allow testing the algorithms to be implemented on custom low-power, miniaturized implantable hardware.

**Figure 2:**
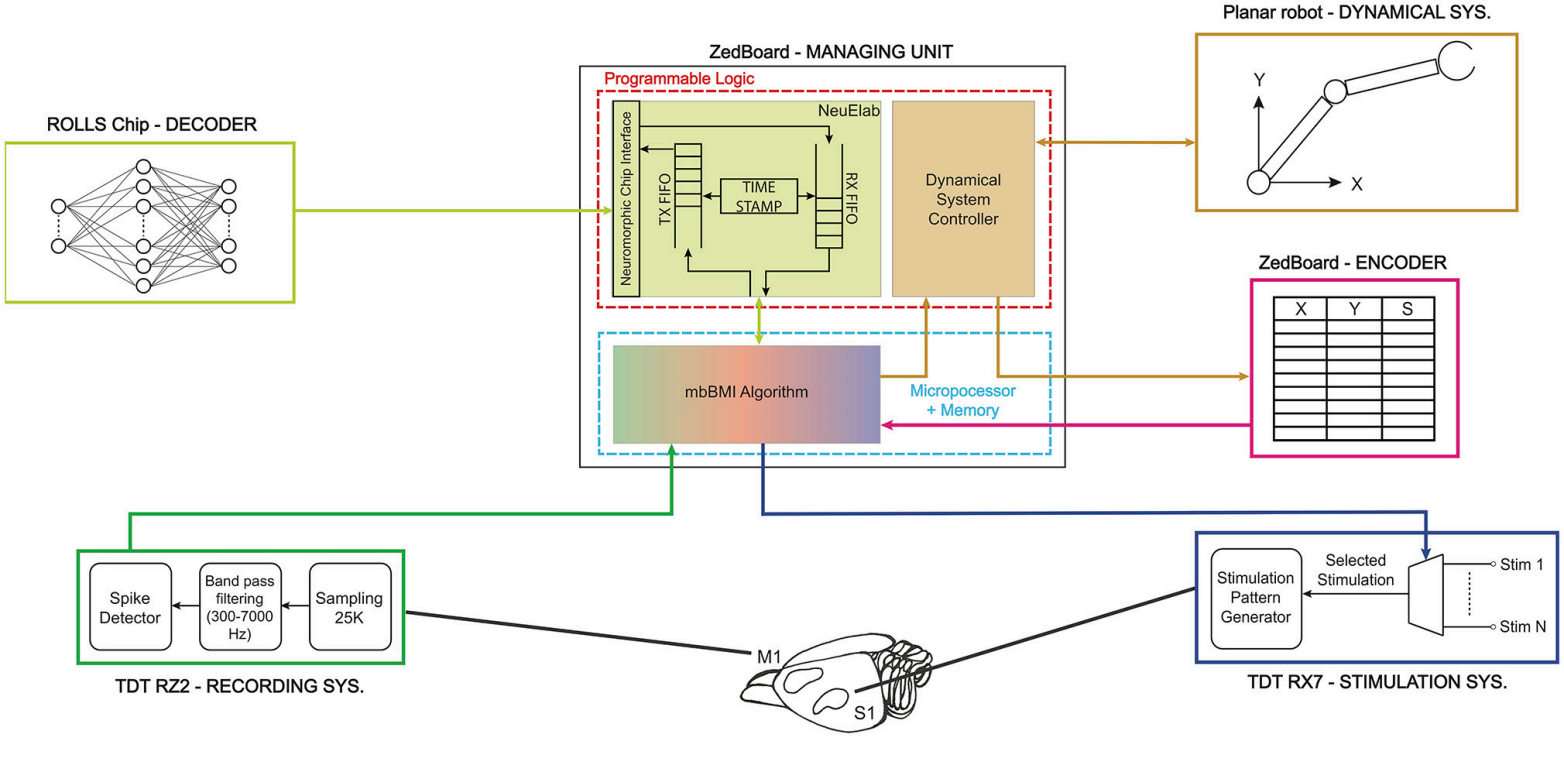
**Real implementation of a modular bidirectional BMI**. The Managing Unit is implemented on a ZedBoard development board that communicates via User Datagram Protocol (UDP) with a *TDT RZ2 BioAmp Processor*—Tucker-Davis Technologies—(acquisition system) and a *TDT RX7 Stimulator Base* (stimulation system). The ZedBoard is connected to the ROLLS neuromorphic processor (decoder) that implements a neural network that is able to learn to decode the neural signal coming from the rat's motor cortex. The decoder's output is translated by the Managing Unit into a two-dimensional force which is converted into digital signals to drive the motors installed on the 2° of freedom robotic device (dynamical system). The dynamical system communicates to the encoder its final state which is transformed into a stimulation pattern that is subsequently delivered by the TDT RX7 into the somatosensory cortex of the subject and closes the loop.

In this implementation, we connected five different satellite modules to the MU realizing the functionalities required by a bidirectional BMI: Acquisition Unit, Stimulation Unit, Decoder, Encoder and Dynamical System, as shown in Figure 2 that have been described in details in Boi et al. (2015a). The Dynamical System (see Boi et al., 2015b) consists of a small mobile cart connected to a water/pellet dispenser mounted on a vertical wall in a custom-made behavioral box for rodents and controlled by two servomotors spanning an area of 38 × 38 cm. The cart is protected by a transparent acrylic glass sheet with a slot that allows the rat to grab the food if the cart is positioned in the desired position. The Dynamical System was designed, developed, and tested in this way to be used in future experimental sessions with behaving subjects.

The main algorithm running on the MU named *mbBMI algorithm* is in charge of reading the spiking neural data coming from the Acquisition System module and communicating them to the decoder. Once the decoder generates an output signal, the algorithm transforms it into motor commands usable by the Dynamical System. To close the loop on the brain, the algorithm acquires the current position reached from the external device and communicates it to the encoder that returns the next stimulus to be communicated to the Stimulation System module.

#### 2.3.1. Managing unit

We implemented the Managing Unit by using the development board ZedBoard^TM^ equipped with a Xilinx Zynq®-7000 All Programmable System On Chip (SoC). The Zynq®-7000 family integrates a feature-rich dual-core ARM Cortex^TM^-A9 based processing system (PS) and 28 nm Xilinx programmable logic (PL) in a single device. In our implementation, the PL runs a custom module that can interface with neuromorphic chips and implements two software modules named NeuElab and Dynamical System Controller. The NeuElab module acquires the pre-processed brain signals from the *mbBMI algorithm* and routes them to the decoder and vice versa, via its hardware interface (Zynq2Neuro described in Section 2.3.1).

The MU stores the temporal offset of each recorded action potential with respect to the last delivered stimulation, as a list of time-stamps associated with the identity (or address) of the emitting electrode. At the end of each recording period, spike trains are generated from the recorded spike time-stamps according to the decoder's requirements (Section 2.5 and **Figure 5**) and then forwarded to the neuromorphic chip. The MU communicates with the decoder using the native neuromorphic asynchronous communication protocol, known as Address Event Representation (AER) protocol (Mortara, 1998), where the information is encoded in the implicit timing between digital pulses (or spikes) and in the identity (or address) of the neuron that has emitted the pulse. The decoder's output AER spikes are acquired by the MU and forwarded to its Dynamical System Controller part.

When acquired on the MU clocked system, the implicit temporal information in the AER spike sequence is explicitly paired with the address of the spike by the TimeStamp block of the NeuElab part of the MU. NeuElab is composed of two different FIFOs that drive the data flow from/to the neuromorphic chip. The TX FIFO is filled with the address of the neuron that shall receive the spike and the time relative to the other spikes, by associating a delay time value by the TimeStamp block. NeuElab reads the TX FIFO and sends a spike to the neuromorphic chip at the time specified by the delay, the address associated to the spike allows the receiving chip to rout the spike to the corresponding neuron. The RX FIFO is filled with the spikes from the neurons of the neuromorphic chip. The received pairs of address and relative time-stamp are then sent to the BMI algorithm that translates the recorded neural activity into commands for the Dynamical System.

Besides managing the AER communication with the neuromorphic chip, the NeuElab interface is critical for the chip's configuration, through digital configuration bits and a number of tunable analog voltages or currents (biases) that set the operating point of the analog circuits. NeuElab can be used, in principle, for interfacing the BMI with any neuromorphic chip that uses the AER communication protocol. In this implementation, the output spiking activity of the neuromorphic chip is translated into a bidimensional force applied to the Dynamical System by means of a pair of Pulse Width Modulated (PWM) analog signals generated by the ZedBoard that drive the external object.

### 2.4. Hardware aspects of the neuromorphic decoder

The decoder that transforms the recorded brain activity into motor commands is implemented on a neuromorphic chip. In the following, we describe the chip and the printed circuit board (PCB) that we developed to connect the chip with the rest of the system.

#### 2.4.1. The ROLLS neuromorphic processor

**Figure 3:**
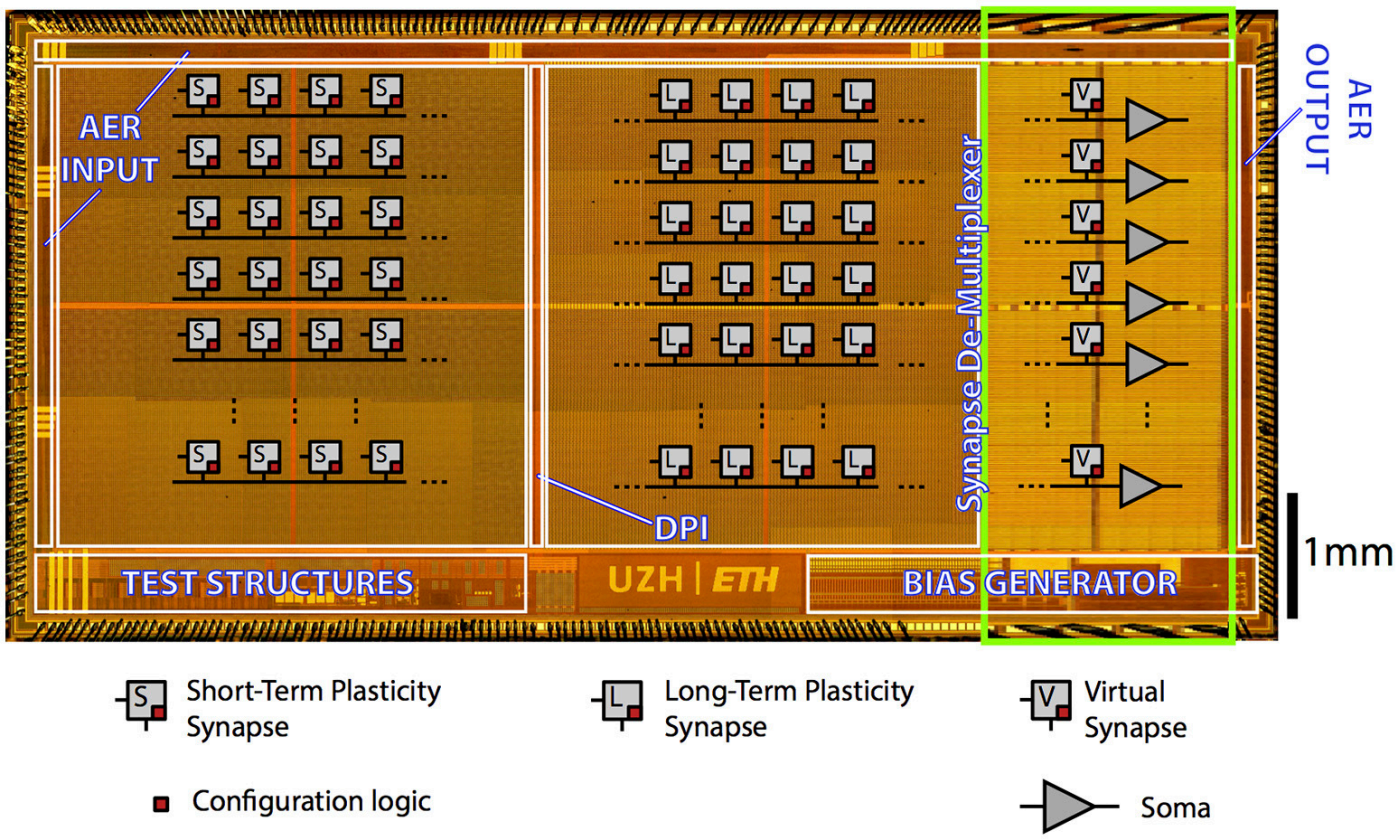
**ROLLS Neuromorphic Processor: micrograph of a neuromorphic processor chip that allocates most of its area to non-linear synapse circuits for memory storage and distributed massively parallel computing**. The test structures in the lower left part of the chip contain extra low power neural amplifier circuits and spike-based neural signal Analog-to-Digital conversion circuits that have not been used in this work.

The Reconfigurable On-line Learning Spiking (ROLLS) Neuromorphic Processor is a general-purpose spiking neural network chip (Qiao et al., 2015). Figure 3 shows the chip micrograph. It was fabricated using a standard 6-metal 180 nm CMOS process, occupies an area of 51.4 mm^2^ and has approximately 12.2 million transistors. It comprises 256 adaptive exponential integrate-and-fire neurons implemented in a mixed signal analog/digital circuit design.

There are 128 K synapses, of which 64 K that can implement a Hebbian plasticity rule (Brader et al., 2007; Mitra et al., 2009) [Long-Term Plasticity (LTP) synapses] (Mostafa et al., 2014). The rest 64 K synapses can exhibit short term depression and short-term facilitation dynamics [Short-Term Plasticity (STP) synapses], and have two possible programmable weights resolution, in addition to the possibility to configure them as either excitatory or inhibitory. These two synaptic matrices (LTP and STP) allow arbitrary on-chip connectivity thanks to a crossbar structure. In principle all-to-all connections are possible through the programmable logic state of the synapses. Additional circuits next to the neurons' array represent the calcium concentration at the post-synaptic side, needed to implement the spike-based LTP weight update algorithm (Brader et al., 2007). We refer the reader to Qiao et al. (2015) for a detailed description of the circuits.

Both the neural network architecture and the parameters of the neuromorphic core are fully programmable via a high-level Python framework (Stefanini et al., 2014). The combination of reconfigurable hardware with the Python-based configuration framework supports the exploration of a wide range of spiking neural network architectures, and their real-time emulation in closed-loop setups. Here, these enabled us to configure a hardware implementation of a spiking neural network that learns on-line to decode patterns of recorded spike sequences.

#### 2.4.2. The Zynq2Neuro (Z2N)

**Figure 4:**
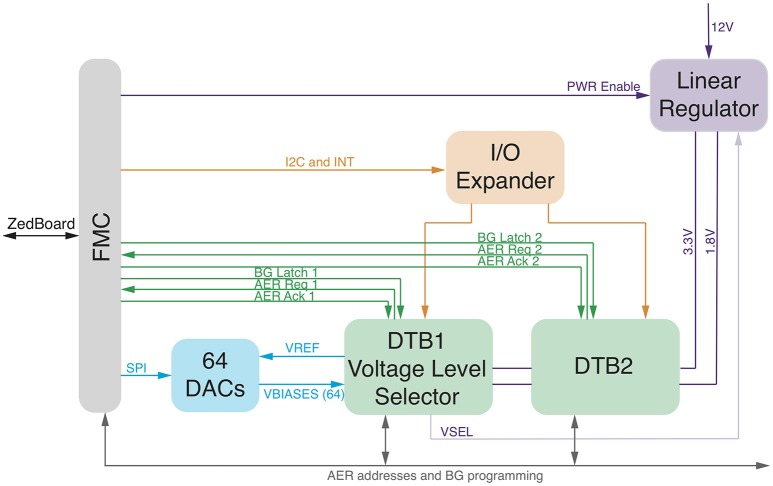
**Zynq2Neuro schematic: block diagram of the board allowing the use of neuromorphic chips in the bmBMI**. It hosts DTBs with neuromorphic chips and connects them to the ZedBoard through the FMC connector. Chip configuration is supported by Digital to Analog Converters or Bias Generator programming and by IO expander for digital configuration. AER input/output communication supports P2P and SCX protocols.

With the aim to manage, program, and interface neuromorphic chips with the Managing Unit, we designed and developed the Zynq2Neuro (Z2N) PCB that can host up to two daughterboards (DTB) that mount neuromorphic chips. The Z2N connects the neuromorphic chips to the FNC connector of the ZedBoard, supplies power to the chips and supports the AER communication and the chip configuration signals. Analog biases that configure the parameters of the silicon neural and synaptic models on the neuromorphic chip can be set either by means of external digital to analog converters (DAC), or by on-chip programmable bias generators (BG) (Delbruck and Lichtsteiner, 2006). NeuElab, together with the Zynq2Neuro board, can drive both systems, the Zynq2Neuro board hosts 64 DACs that can be programmed through an SPI interface and also hosts the necessary signals for programming different types of BGs, managed by NeuElab, hence supporting a large library of neuromorphic chips. The Z2N board is already configured to support future chip functionalities by means of I/O expanders and I^2^C protocol. The AER addressing space can be expanded up to 30 bits (configurable as inputs or outputs). The Z2N (Figure 4) can support logic levels, power supply and biases from Digital to Analog Converters of 3.3 or 1.8 V, as selected from the first DTB. This means that the two DTBs need to host chips that are homogeneous for the logical levels. In general, the Z2N can support chips fabricated on the 350 nm (3.3 V) and 180 nm process of the latest generation (1.8 V and mixed 1.8/3.3 V). To optimize the design, AER address lines, some bits of the Bias Generator programming, I^2^C and I/O expander are shared among the two DTBs. The sharing of the AER address lines is based on the assumption that they are in tri-state when the chip is not sending or receiving an event. This is guaranteed by the SCX protocol (Mortara, 1998), but can be supported also for the P2P protocol (Boahen, 2000), by adding buffers on the DTB driven by the handshake signals (ACK) from the ZedBoard. The correct addressing of the event to/from the chip is guaranteed by the reserved handshake signals (REQ/ACK and Bias LATCH) that target only one of the two chips. The Z2N specifically targets compatibility with neuromorphic chips such as the ROLLS (Qiao et al., 2015), but is a more general tool for most of existing neuromorphic chips based on parallel (or word-serial Boahen, 2004) AER protocols, on Bias Generators externally configurable by means of SPI-like serial interfaces, or on external voltage tuning. Some examples of supported chips are the Dynamic Vision Sensor (Lichtsteiner et al., 2008), the AER EAR (Chan et al., 2007), the Selective Attention Chip (Bartolozzi and Indiveri, 2009), the spiking Winner-Take-All chip (Chicca et al., 2014), and the Asynchronous Time-Based Image Sensor (Posch et al., 2010).

### 2.5. Algorithmic aspects of the neuromorphic decoder

We approached the neuromorphic decoding task by combining the constraints of a multi-class classification task with those of spiking neural networks with limited resolution synaptic weights, and with the BMI-specific requirements related to the simultaneous contribution of all four classes to each decoded force (see Section 2.1).

#### 2.5.1. The silicon spiking neural network

We configured the ROLLS chip to implement a feed-forward spiking neural network that exploits the spike-timing dependent plasticity of the chip's LTP synapses to learn how to extract the pattern of four calibration forces that should result in the net desired force, from the recorded neural activity. Each of the output neurons of the network was trained to act as a binary classifier by re-weighting the features of its input that were distributed across its synapses, so as to ultimately yield, via its activation function, a higher output spike rate for one, positive class of input compared to the other three, negative classes. Neurons were grouped into four ensembles, each corresponding to one of the four stimuli. The spike counts output by the four ensembles during the presentation of the recordings to the network were directly used as the coefficients that weight the contributions of the four component forces acting on the BMI's end effector.

#### 2.5.2. Mapping the neural recordings to the ROLLS neuromorphic processor

The spike-based learning algorithm implemented on the chip is based on the model proposed in Brader et al. (2007). Using this model, feed-forward neural networks can learn to classify patterns based on their mean rates. However, in the neural data we recorded, the principal feature that distinguishes one class from the others is the precise timing of the recorded spikes, aligned to the offset of the sensory micro-stimulation (Figure 1). Therefore a transformation of the input spike sequence into an array of firing rates is required before it reaches the output layer. Furthermore, the number of non-redundant features in the data needs to be sufficiently high to support robust discrimination across all classes, but the recorded activity was very similar across all recording channels (see Figure 1, inset). Therefore it is likely impossible to find a single-layer feed-forward network configuration that can classify the recordings based on features corresponding directly to the recording channels.

**Figure 5:**
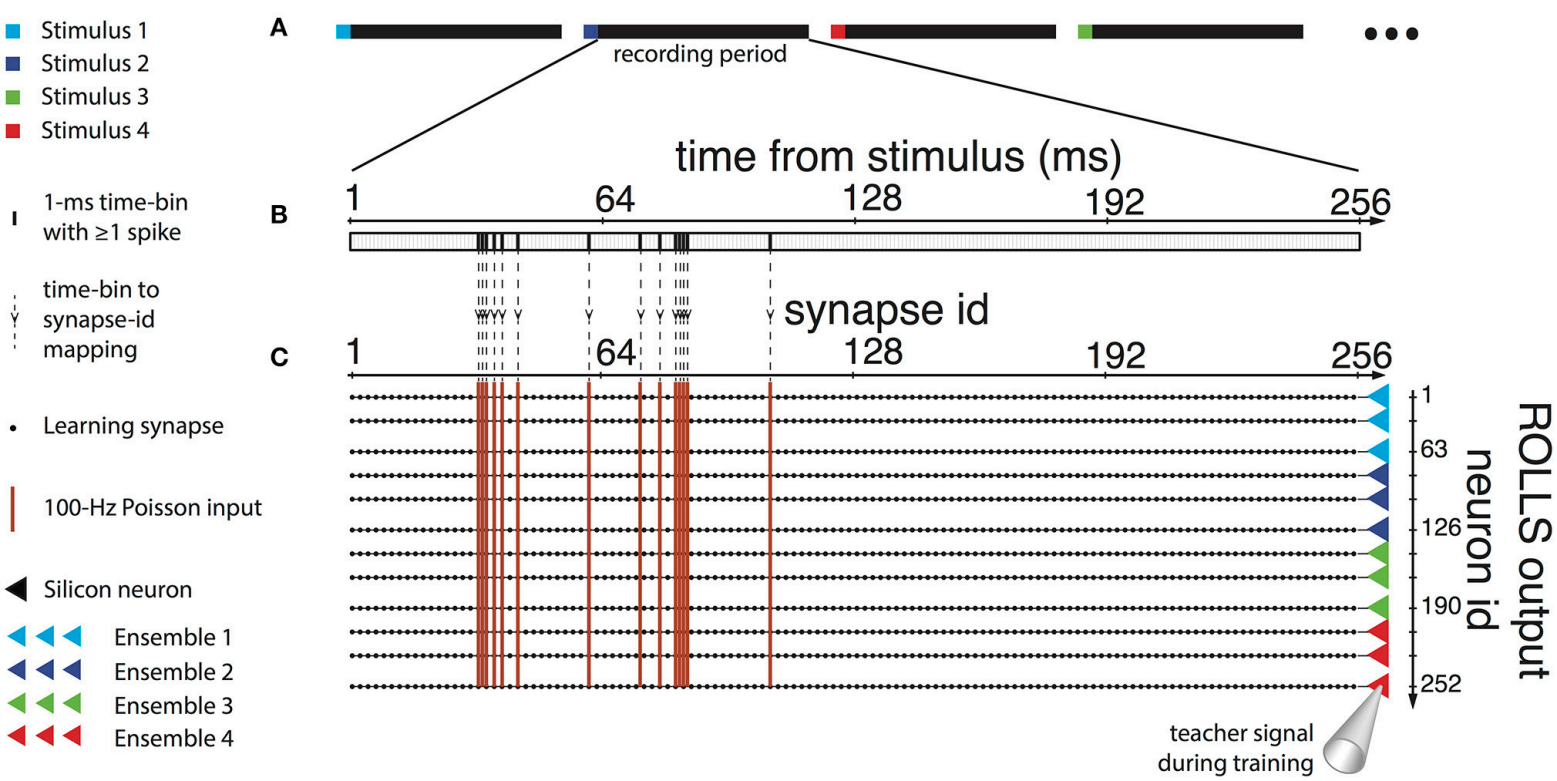
**Input, training, and use of the neuromorphic decoder**. **(A)** To train the decoder, four different stimuli were provided to the rat's sensory cortex. Stimuli were provided in random order, 40 times each, and the activity in the motor cortex was recorded during the session. **(B)** The activity in the first 256 ms *after* the end of each stimulus was used with the decoder. The recording was binned in 1 ms time bins, and bins where at least one action potential was detected across any of the recording channels were marked. **(C)** Each time bin was mapped to a column of 252 learning synapses on the ROLLS, whereby each synapse belonged to a different post-synaptic neuron on the chip. Synapses corresponding to time bins in the recording that included detected spikes received a Poisson spike train with a mean rate of 100 Hz. Synapses corresponding to empty time bins received no input. In addition, the silicon neurons were stimulated by a teacher signal, as follows. The 252 post-synaptic neurons were separated in four ensembles of 63, and we associated each ensemble with one of the four stimuli provided to the rat's sensory cortex. During the presentation of each recording to the chip, the ensemble corresponding to the preceding cortical stimulus was stimulated by a Poisson spike train of 75 Hz as a teacher signal, while the other three neuronal ensembles received a teacher signal of 25 Hz. After training, the ROLLS received no teacher signal, and each recording was decoded into a force applied to the end effector, by weighting four force components by the number of spikes output by each of the four ensembles.

To reconcile the characteristics of the data with the network requirements we mapped uncorrelated sub-samples of the spike sequence to different synapses of the classifier neuron, using a mean-rate encoding. Specifically, we binned the recorded spike trains in time bins of 1 ms (Figure 5B) and associated each bin with one input synapse of each neuron of the network (Figure 5C). We provided a 400 ms high mean-rate (100 Hz) Poisson spike train to the learning synapses for time bins that contained recorded spikes, and no input to the rest of the synapses (Figure 5C).

Under the constraint of a finite number (256) of available synapses per neuron, there was a trade-off among the number of recording channels, the duration of the recording patterns, and the temporal precision desired. The first 200–300 ms of each recorded pattern included significant differences across the four different classes (Figure 1), that would potentially be sufficient for the classifier to discriminate between them. Based on this, together with the observation that the distributions of spike timings were very similar across different recording channels, we merged the 15 recording channels into a single spike train, and we used the first 256 ms of the recordings, thus acquiring a temporal precision of one ms per time bin. Longer recording duration with a two-millisecond or lower precision was found to diminish decoding performance.

#### 2.5.3. The neural network's task

The aim of the BMI is to best approximate the desired force field over the duration of the experimental session, through weighting the four force components. To achieve this aim, there are two criteria based on which the decoder has to simultaneously optimize its learning. Firstly, it needs to learn to classify the patterns, i.e., to correctly output the single class to which each presented recording truly belongs, as expressed by the “winning” (i.e., the most firing) ensemble of output neurons. Secondly, the decoder also needs to prevent the other three “losing” ensembles from biasing the force field toward particular directions on average over the trajectory of the end effector. That is, it needs to classify the recordings under the constraint of learning to equalize the average outputs of “losing” ensembles. Thus, despite the similarities to a classifier, classification of individual recordings is only partly the decoder's task.

#### 2.5.4. Biased similarities and differences between classes of recordings: addressing them with heterosynaptic competition

The decoder had to address certain additional characteristics of the recordings to achieve its goal of approximating the desired force field over the experiment's course. Specifically, different classes of recordings differed in number of recorded spikes on average, and this difference in the input energy could be reflected as a bias in the chip's output and consequently in the direction of the decoded force in each trial. Moreover, even though spike timing was the principal difference between recordings of different classes, some spike timings were common between classes. This increased the difficulty in distinguishing between different classes. That is, the different classes had a certain level of overlap between their features, which could increase classification errors. Additionally, this overlap was not of the same extent for all pairs of classes, i.e., some classes were more similar to some than to others in terms of common spike timings (Figure 1). This asymmetry could result in additional biases in the weighting of the force components by the decoder, thus misshaping the resulting force field in certain parts of the working space.

To address these points, we used the “stop learning” feature of the ROLLS chip learning circuits (see Brader et al., 2007) which prohibits potentiation of synapses when the post-synaptic firing rate exceeds a threshold. When a certain number of synapses that correspond to a neuron's positive class are potentiated, the increased excitation from the input causes the neuron to stop learning. This introduces heterosynaptic competition (Royer and Paré, 2003) to the chip's output neurons, which serves (a) to normalize the network's output in response to different classes, (b) to make potentiated synapses a scarce resource hence biasing potentiation toward non-overlapping features, and (c) to equalize the output of “losing” ensembles. In addition, combined with device mismatch on the neuromorphic circuits, it biases different members of each ensemble to learn a slightly different decision boundary. This is similar to boosting techniques employed in machine learning and improves the classification performance by allowing for non-linear decision boundaries for the ensemble through the aggregation of the multiple linear boundaries defined by the ensemble's member neurons.

#### 2.5.5. Training the neuromorphic decoder

To train the neuromorphic decoder, we used an experimental session composed of 40 repetitions of each stimulation pattern (i.e., 160 evoked recordings). During the training procedure were randomly interleaved (Figure 5A) and presented to the ROLLS processor the 160 training trials, according to the method described in Section 2.5.2 (Figures 5B,C), along with a teacher signal representing the label of the presented example, i.e., the type of sensory microstimulation that produced the recorded neural response. Sixty-three output neurons were assigned to each class (Figure 5C, right). The teacher signal biased each neuron to be tuned to one class, by causing it to fire with a rate that maximized the probability that the neurons synapses got potentiated when an example of that class was presented, and depressed when an example of the other classes was presented. The mean rate of the Poisson spike train that would act as a teacher signal with these properties, as well as the analog parameters of the silicon neurons and synapses of the ROLLS processor were configured to match the characteristics of the input data with the requirements of the learning and of the decoding task.

### 2.6. Assessing the BMI's performance

Once the decoding and encoding interfaces were properly calibrated, in order to test the system we ran the BMI by decoding from each neural trial a bidimensional force and by encoding each position of the controlled object through an ICMS pattern. We used a test dataset of neural recordings acquired by 10 repetitions of each of the four stimulation patterns (i.e., 40 evoked recordings), which were unseen by the BMI during its training. We selected eight different equispaced and equidistant positions as starting points in which the dynamical system was initialized and we ran the BMI 100 times starting from each initial position by obtaining 800 trajectories. We tested the system under two conditions: under normal operation (encoder-ON condition), each test recording was selected according to the dynamical system's current position. An alternate condition (encoder-OFF) was used to test the bidirectionality of the BMI and the learned coordination between the encoder and decoder modules. In the encoder-OFF condition, each test trial was randomly selected among all 40 test recordings.

To assess the repeatability, the speed and the optimality of the generated trajectories we measured the number of steps required to converge to the target and the mean *within-trajectory variance* (abbreviated to *wtv*). In particular, each trajectory's *wtv* is defined as $\sqrt{C_{x}^{2}+C_{y}^{2}}$, where *C*_*x*_ and *C*_*y*_ is the covariance of the distribution of the per-step displacement along the *x* and the *y* axis, respectively. We obtained the mean *wtv* by averaging the *wtv* computed for each set of trajectories that started from one initial position.

## 3. Results

### 3.1. Decoding performance

**Figure 6:**
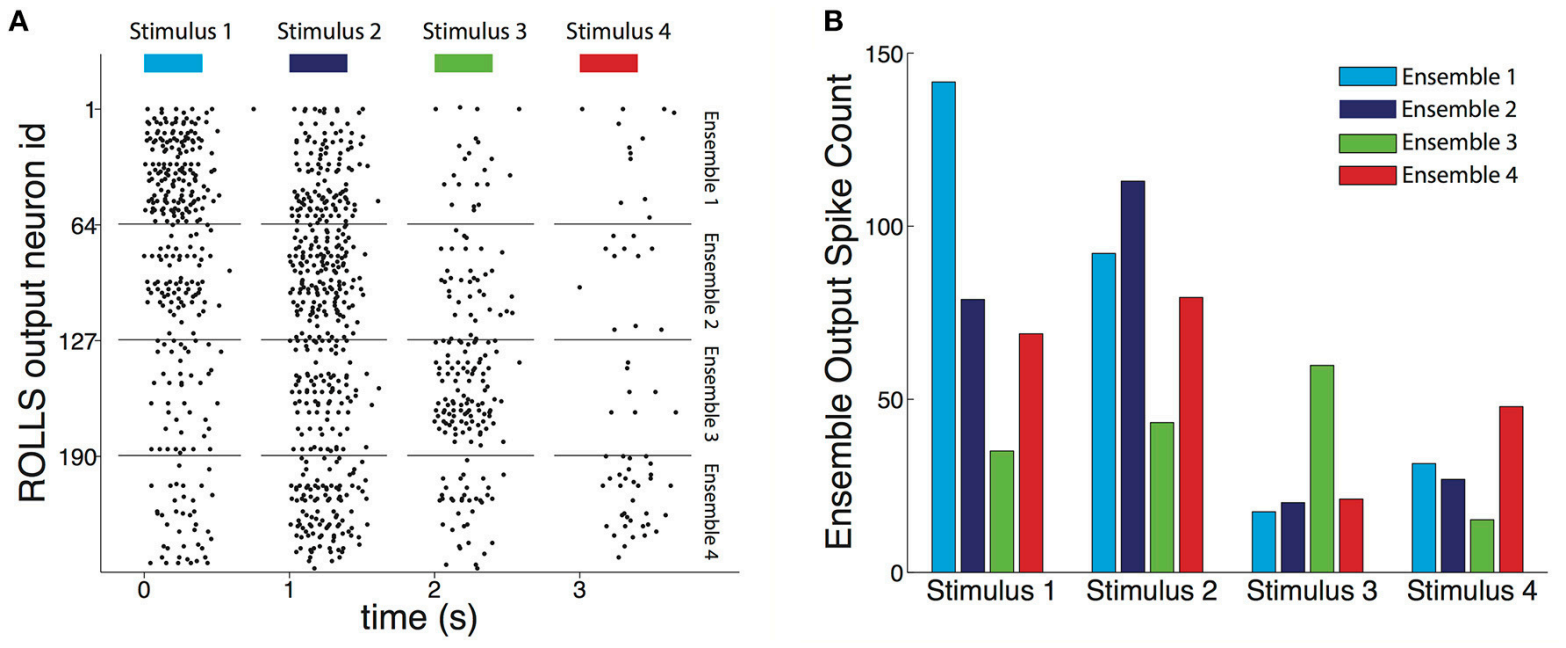
**Output of the trained decoder**. **(A)** Raster plot of the output spikes of the trained ROLLS chip during presentation of four example test recordings each resulting from a different type of stimulus. The length of the bars on top shows the 400 ms long presentation of the input. During presentation of the four examples, the most active ensemble of output neurons corresponds to the true stimulus that caused the input recording. The spike count of the output each of the four neuronal ensembles was directly used to weight each of the four components of the force field to result in the motor command, i.e., force, that acted on the controlled object. The chip's neurons maintained some activity till shortly after the input stopped, mainly due to excitatory current leaking between the firing neuronal electronic circuits. **(B)** Average output spike count for each ensemble of neurons, for each type of stimulus that caused the decoded recording. For each stimulus, its corresponding ensemble fires on average more than the other three, demonstrating the classification aspect of the decoder's task. In addition, the decoder learned for each stimulus to partially equalize the response amplitudes of the three non-corresponding ensembles, compared to the extent of the differences between input classes (cf. Figure 1 and see Section 3.1).

To assess the decoder we used test datasets, which were previously unseen by the decoder, as described in Section 2.6. For each decoded pattern, the output spikes produced by each neuronal ensemble (Figure 6A) were counted. Given a stimulus, the average spike count of the ensemble of silicon neurons corresponding to that stimulus was higher than the other three (Figure 6B).

In addition, as a result of the introduction of “stop learning” to the silicon neurons average spike counts were relatively uniform across the other three ensembles despite the biases in pairwise similarities between input classes (see Section 2.5.4). The chip learned to suppress this bias, and, consequently, decoded resultant forces for each stimulus were, as originally intended, most similar to one of the four forces used during the calibration phase (colored thick arrows shown in **Figure 8B**).

While the task of the decoder was not a pure classification task and it was not optimized to perform as a classifier, we also evaluated its performance in correctly classifying the recordings, as expressed by the maximally firing ensemble of neurons. For 20 different random splits between the training and the test sets, the classification performance on the test set ranged between 50 and 70% correct, with the chance performance level being at 25%.

### 3.2. BMI performance

**Figure 7:**
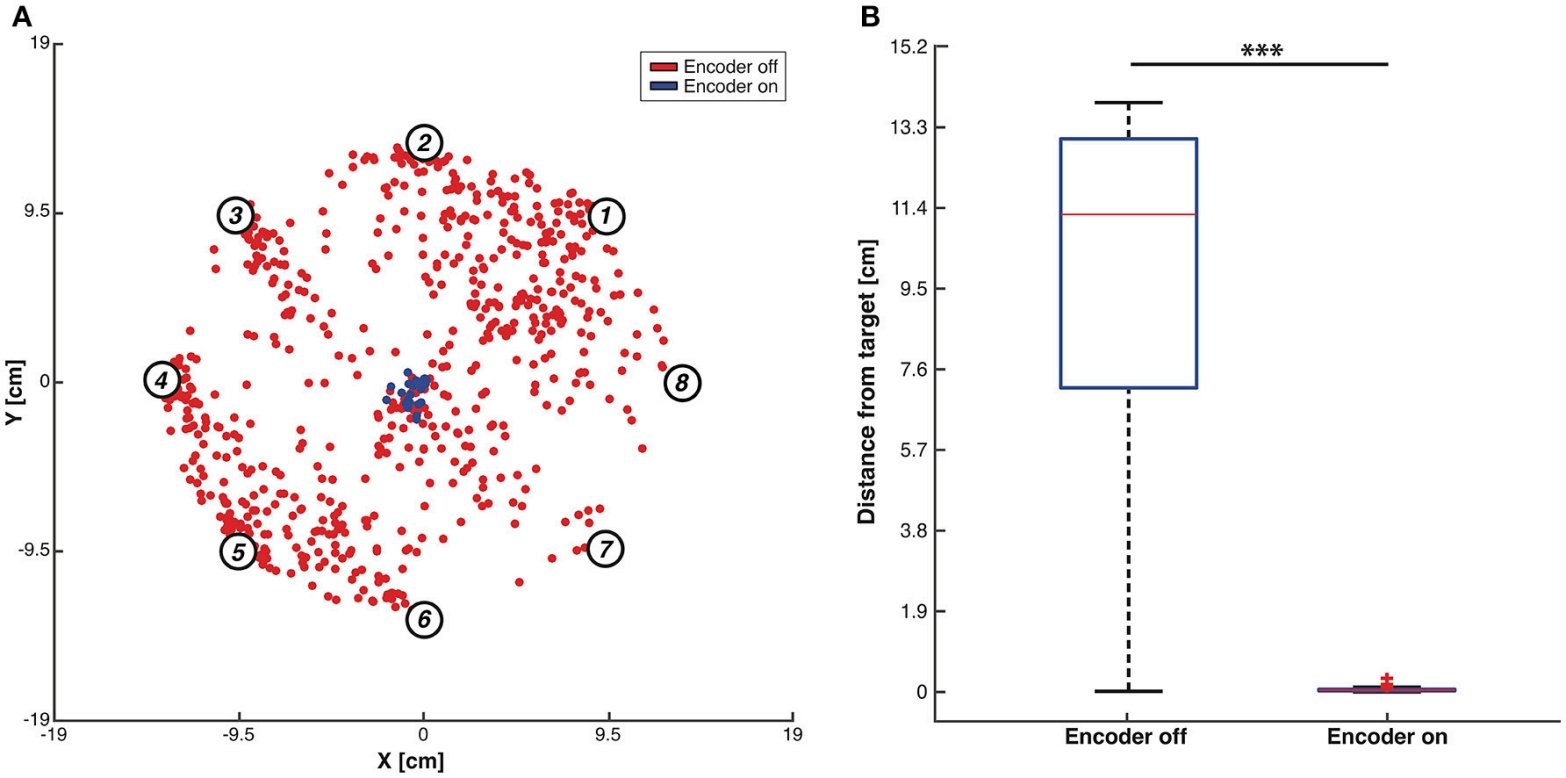
**Testing of BMI performance with 100-steps stopping rule**. **(A)** Trajectories closest points to target. Red dots indicate, for each trajectory, the closest points to the workspace axis origin with the encoder switched OFF while blue dots represent the same points for the trajectories generated with the encoder ON. Data were collected by running the BMI 100 times for each of the eight predefined initial positions (i.e., numbered circles) both with the encoder turned ON and OFF. **(B)** Box plots of the trajectories closest points distributions with the encoder ON and OFF. Two-sample *t*-test, ^***^p < 0.001.

In order to assess the BMI performance, we performed two different testing sessions: during the first session we set the maximum number of steps to 100 as stopping rule for the obtained trajectories (Figure 7). The BMI moved the object freely according to the sequence of forces that the closed-loop set-up applied and we placed the target as the origin of the axes. In each trial, the controlled object was initialized at one of eight starting positions and the BMI generated one trajectory of 100 encoding + decoding steps. We marked and plotted in the figure the point that was closest to the origin of the axes considered as the target point (Figure 7A). For each starting position we repeated the experiment 100 times, yielding 800 points in each of the two conditions (blue points for “Encoder ON” and red points for “Encoder OFF”). In condition ON, when a stimulus was provided to the sensory cortex, it was according to the current position of the object. In condition OFF, the stimulus was selected randomly among the four possible stimuli, thus not encoding the current position of the object. The distributions of the two sets of points (Figure 7B) are statistically different (independent samples *t*-test, *p* < 0.001) showing a decrease of 99% in the distance from the target and demonstrating that closing the loop in the proposed BMI is crucial in order to correctly drive the dynamical system toward a target.

In the second testing session, we simulated a real experiment in order to generate motor commands that drive a mobile cart from predefined initial positions toward a target position represented by a slot in the glass that allows the rat to get the reward (Boi et al., 2015b). In this session to distinguish between convergent and non-convergent trajectories, we defined the target as a circular region with radius set to 3.6 cm placed in the center of the workspace. A trial was considered successful as soon as the generated trajectory reached the borders of this area. When this happened the BMI was disconnected and the cart was automatically positioned in the center of the slot to allow the subject to receive the reward.

**Figure 8:**
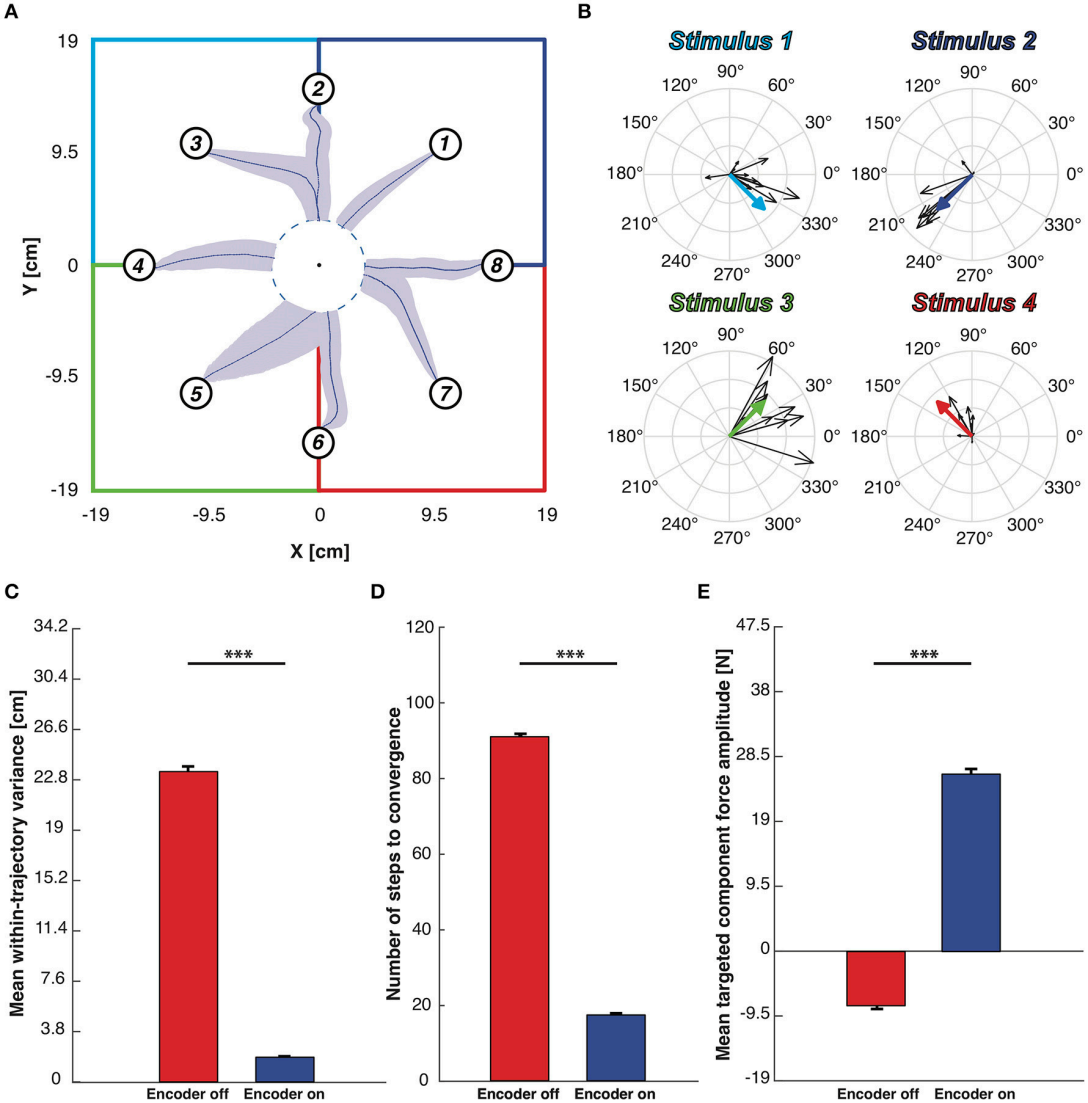
**Testing of BMI performance with target-region stopping rule**. **(A)** Mean trajectories plot. Starting from each starting point depicted with a numbered circle, the blue lines represents the mean trajectories and light blue areas represent the covariance of the trajectories along 100 trials. The workspace is subdivided into four sensory regions, one per each stimulation, highlighted by four different colors. We defined a target region centered in the origin of the axes and whenever the mobile cart reaches its edge the BMI considers the task accomplished. **(B)** Black arrows represent the decoded forces computed during the BMI test phase. Colored thick arrows represent the four calibration forces associated to the sensory regions. Forces were grouped on the basis of the stimulus that generates them. **(C)** Mean *within-trajectory variance (wtv)* ± SEM of all the 800 trajectories recorded both with the encoder turned ON (blue bar) and OFF (red bar). **(D)** Mean number of steps to convergence ± SEM. The red bar, obtained with the encoder turned OFF, is quite close to the maximum step allowed (100 steps) while when the encoder is active the steps number necessary to reach the target region is significantly lower. **(E)** Mean *DT* component magnitude ± SEM. Each decoded force has been split into *Directed to the target - DT* (magnitude of the force that points toward the target) and *Orthogonal to the target - OT* one (part of the force perpendicular to the directive component). The mean magnitude of the DT component obtained from forces generated with the encoder turned off (red bar)is much higher than when the encoder activated (blue bar). Two sample *t*-test, ^***^p < 0.001).

Figure 8A shows the mean trajectories (blue lines) and the covariance (light blue area) generated during this experimental session with the encoder turned ON. Two distinct behaviors are distinguishable (see Figure S1C): if the pathway from the starting position to the target region lies inside the same sensory regions, we obtained an almost straight trajectory. On the other hand, when the controlled device crosses the border of one region, the systems oscillates along the border of the two adjacent regions. This particular behavior does not represent a decoding error but rather reflects the limitation of having only four different stimulation patterns encoding the information about the region in which the device is, disregarding the precise position inside it (Tehovnik, 1996; Romo et al., 1998). The BMI converges to the target region with a 100% success, and it does so in a very stable and straight path because the decoded forces obtained in response to the same stimulation pattern are very similar to each other, both in terms of direction and magnitude. This is demonstrated in the compass plots in Figure 8B showing that the forces decoded from the neural activity evoked from each stimulation pattern and used during the testing phase (i.e., black arrows) are almost overlapping. In order to further assess the neuromorphic decoding capabilities we also report the forces used to calibrate the BMI motor interface (colored thick arrows in Figure 8B) that, especially in terms of direction, are almost equal to most of the related forces decoded during the BMI run. In the encoder-ON case the mean *wtv* and the steps needed to reach the target region significantly decrease (respectively, 92 and 80%) with respect to the encoder-OFF case (Figures 8C,D).

Finally, for each force produced by the decoding process, we measured the magnitude of two components: the component of the force that points toward the target point, named *Directed to the target—DT*, and the component orthogonal to it, named *Orthogonal to the target—OT*. The mean of the DT-component is strongly positive (directed to the target) in the case of encoder-ON and slightly negative (divergent from the target) when the encoder is turned OFF (Figure 8E shows an increase of 69%). In both conditions (ON and OFF), the mean OT-components are almost null compared to the mean *DT* obtained with the encoder-ON (respectively, 90 and 97% less). In the OFF condition, this can be attributed to the randomness of the motion. In the ON condition, combined with the increased DT force, this is an indication of successful decoding.

Figures S1A,B show the complete set of trajectories collected without using the target-region stopping rule, respectively, with the encoder switched ON and OFF. Figures S1C,D shows the set of trajectories used to build the different panels of Figure 8.

## 4. Discussion

In this paper, we showed the applicability of neuromorphic hardware in a brain-machine interface system, in the first demonstration of this kind. In particular, the decoder module of the BMI was implemented by a spiking neural network on a mixed-signal analog/digital neuromorphic processor, the ROLLS, that learned to perform on-line the decoding of the neural recordings into commands that addressed the brain-controlled device.

The analog neuromorphic circuits of the ROLLS neuromorphic processor emulate functions of biological neurons and synapses by replacing biophysical properties with analogous properties of the sub-threshold physics of transistors. The resulting spiking neural networks operate on a power-efficient and compact system for applications of pattern recognition such as a BMI decoder's task. On the other hand, because of these underlying principles of operation, analog neuromorphic circuits like the ones found on the ROLLS are imprecise and variable, similar to biological neural elements, in sharp contrast to simulations of spiking neurons and synapses on digital neuromorphic or general-purpose processors. The neuromorphic decoding task was further complicated by the variability in the recorded data, and by the overlap in spike-timings between the to-be-discriminated classes.

Further difficulty arose by the fact that the decoder's task was not a standard classification task, as the BMI required the decoder to output a contribution of all potential classes of recorded activity simultaneously, while preventing the average chip output from being biased toward any pair of classes, even though the pair-wise similarities between classes were biased.

Despite these particularities, the spiking network we designed successfully learned the decoding task, enabling the BMI to perform at similar levels of a previous non-neuromorphic version of the bidirectional BMI. This was achieved by exploiting two key characteristics of the ROLLS chip: variability between silicon synapses and neurons deployed into an ensemble learning technique that aggregated multiple weak classifiers into a powerful one, and the heterosynaptic competition through the “stop-learning” feature of synapses on the ROLLS chip, which enabled the network to focus on the discriminative features of the input, thus both improving classification performance and reducing the reflection of biased similarities in the input onto the output of the trained network. A key feature of the decoder is that the spiking output of the neuromorphic chip is directly used to compute the force controlling the end-effector. The components of the force were weighted by the spike counts of the chip's output, an important step toward using neuromorphic hardware not only as a decoder, but also as prosthetic controller.

### 4.1. Features of the proposed neuromorphic decoder

The set-up we propose has been designed as an initial proof of concept prototype to evaluate the potential of neuromorphic hardware computing in BMIs, and to determine its limitations; within this context, this work shows that, even at this level, integration of neuromorphic hardware in set-ups characterized by the intricacy of a bidirectional BMI is technically possible. Our results show that, despite the low precision, low resolution, and noisy (but compact and low-power) analog electronic circuits in the neuromorphic chip, the system built in this way can recognize multi-dimensional input patterns. In particular, the results demonstrate how this neuromorphic hardware can be configured to produce the correct average forces over the controlled object's trajectory (Figure 8A), despite the fact that the forces decoded from individual recordings could strongly deviate from the target (Figure 8B) due to the contributions of all four force components combined with unbalanced inputs (Figure 1). A unique aspect of the specific neuromorphic hardware used is represented by its ability to learn these computationally demanding tasks, with on-chip real-time spike-based plasticity circuits, as opposed to learning the network parameters off-line and configuring them at run-time. The flexibility provided by the digital event-based communication infrastructure, and the digital registers embedded in the chip, next to the subthreshold analog neuromorphic circuits, allow this system to be used in a variety of tasks that require real-time decoding or classification of sensory inputs, or real-time encoding of desired outputs. Although, the analog circuits have time constants of the order of milliseconds (in order to provide biological realism, and importantly, to minimize power consumption), the real-time response properties of the chip at network level have latencies that are extremely small (e.g., below tens of microseconds). This allows the chip to decode the neural activity on line in the BMI's loop, within one time step of the dynamical system's operation, whose bottleneck is determined not by the decoder, but by the inter-stimulus interval. The average power consumption of the chip, which has been measured to be approximately 4 mW, is competitive with state-of-the-art DSPs and much lower of general purpose low-power computing units that could be used to run the pattern recognition software. It is worth noting however, that since in the current set-up the neuromorphic chip is interfaced to additional devices mainly used for prototyping and debugging, the overall system requires additional relatively high power and area.

### 4.2. Limitations of the system and proposed future additions

The simplicity of the single-layer feed-forward network of only 252 neurons that was employed for this particular application demonstrates the limitations and computational power of physical instantiations of spiking neural networks and suggests that further development of analog neuromorphic hardware and spike-based algorithms may yield a computationally powerful, yet low-power consuming alternative to software and conventional processors for a broad spectrum of tasks. With respect to the neuromorphic BMI decoder in particular, further work could enable two specific improvements and additions.

Firstly, the present implementation addresses the complex temporal dynamics of the recordings with a processing step introduced between the neural recording and the output layer of the neural network, and performed off-chip, which transforms the temporal dynamics of the recordings to a spatial pattern input to the chip. While the method proposed is suitable for the presented system, we have been investigating alternative algorithms and spiking neural network architectures that can potentially decode and recognize these types of spatio-temporal patterns entirely on the chip. In this way, the chip could directly receive the recorded spike train, and operate on it with no need for an intermediate off-chip storage step. This would be possible because of the ROLLS' real-time operation, with time constants that match those of real neurons. To this direction, Corradi and Indiveri (2015) perform a binary classification task on spatio-temporal recordings from the zebra finch, using reservoir computing on the ROLLS' silicon neurons, which demonstrates that future development of these types of methods can permit their application on a BMI.

On a separate but related note, here the BMI operated in discrete time steps. This permitted us to insert the processing step that inputs the recorded spike timings as rate-coded patterns into the ROLLS chip, without loss the system's continuity. Nonetheless, this will be a crucial obstacle for the decoding module's integration in future continuously operating BMIs. On the other hand, the limitation does not originate in the ROLLS chip itself. The chip does not have an internal clock that must be synchronized with the chosen time points. It rather recognizes inputs in which time represents itself in the spike train's statistics. This implies that removing any off-chip transformation that intermediates the input would also enable the on-line use of the chip in continuous-time BMI set-ups.

As a further future improvement, the fact that the network learns on line could be used to allow the decoder to adapt to changes in the neural responses with time. Specifically, in the current implementation, the decoder updates itself incrementally after the presentation of each training pattern. Training inputs are combined with a teacher signal that biases different neurons to strengthen or weaken their connections to different features of the input, through imposing different levels of output firing during the presentation of different input classes. After training, we use the chip to decode new recordings of brain activity. The on-line learning feature is not crucial for demonstrating the performance of the BMI in its current instantiation, but can become useful in future chronically implanted setups, that have to adapt to continuous slow changes in the nature of the signals being recorded. In such a future implementation, learning could continue during the chip's use as a trained decoder. As the trained silicon neurons respond with high firing rates to their corresponding input classes, and with lower rates to the other classes, the neurons could bias themselves to continue correctly adapting their synapses to the input patterns in the absence of an externally provided teacher signal. This would be made possible after tuning the parameters of STDP synaptic dynamics of the ROLLS to enable potentiation and depression in the ranges of firing rate that the trained neurons output when decoding the input.

### 4.3. BMI modularity

As technological and scientific progress accelerates, it brings new opportunities for improving the quality of life of millions of people. The interdisciplinary field of brain-machine interfaces largely relies on the rapid evolution in the diverse fields that are involved (Nicolas-Alonso and Gomez-Gil, 2012). Nevertheless, the complexity of BMI systems, the interdependence of their components cause them to be very difficult to manage, test, modify, and upgrade. Our work suggests a possible solution to this issue by proposing a new modular implementation that allows to modify or update each module without changing the entire system.

The modularity allows to develop different parts of the BMI in different labs and assemble the complete system by plugging in these parts as modules. This structure makes easier and more reliable both the implementation of the single module and its integration in the complete system. Parallel development of components could also accelerate the ultimate realization of a device compact and powerful enough to be used as clinical tool able to transfer data between the brain and external devices wirelessly through an implanted interface (Azin et al., 2011; Fan et al., 2011; Borton et al., 2013; Angotzi et al., 2014). In this work, we also demonstrated that the modular architecture does not affect BMI performances, showing results comparable with the ones achieved in Vato et al. (2012); this result suggests that BMI systems developed in other labs could also be re-implemented in a modular manner. To help the interested scientist in doing this, most of the material used in this project is freely available on Si-Code website : http://www.sicode.eu/results/software.

## 5. Conclusions

The relevance of neuromorphic technology in the design of brain-machine interfaces is demonstrated by the flourishing work in this domain (see Dethier et al., 2013; Barsakcioglu et al., 2014; Hogri et al., 2015, as non-exhaustive examples). The main features of neuromorphic implementations are low power consumption, real-time operation, adaptability, and compactness. Simulations show that hardware Spiking Neural Networks can successfully decode the activity of neurons for closed-loop cortical implants (Dethier et al., 2013) and an ad-hoc working prototype is able to substitute a cerebellar learning function in the rat (Hogri et al., 2015). Our work extends this approach in proposing a modular and reconfigurable scheme whereby the neuromorphic chip can be exploited for implementing different algorithms and BMI functions; in particular, we demonstrated this approach by using the chip as neural decoder. We also explored the impact of using a neuromorphic decoder in such a closed-loop system by comparing its performance with the one previously developed in our lab.

As in Vato et al. (2012), we closed the loop with the brain by decoding the neural activity evoked by different patterns of intracortical micro-stimulation selected by the encoder. Even if we are not decoding from the anesthetized subjects any volitional input, this system, establishing a bidirectional interaction between the brain and an external device, needs to be considered the first necessary step toward the design of future experiments involving behaving subjects controlling the movements of a small mobile cart connected to a water or food dispenser (Boi et al., 2015b). The unique characteristics of the neuromorphic decoder will allow our modular bidirectional BMI to integrate the volitional component of brain activity in the decoding scheme and to explore the integration of the volitional input with the automatic brain response in controlling the movement of the external device.

## Author contributions

FB, VD, FD, and CB designed, built, and debugged the hardware and software infrastructure for the mbBMI. TM and GI implemented the spike-based learning and decoding algorithm on the ROLLS neuromorphic processor. FB and AV performed the experiments and collected the neural data. FB, VD, and TM performed all the analysis presented in the paper. All the authors contributed in writing and editing the manuscript.

### Conflict of interest statement

The authors declare that the research was conducted in the absence of any commercial or financial relationships that could be construed as a potential conflict of interest.
