# Supplementary material for: A Bidirectional Brain-Machine Interface Featuring a Neuromorphic Hardware Decoder
Source: Front Neurosci. 2016 Dec 9;10:563. doi: 10.3389/fnins.2016.00563 (PMC5145890; doi:10.3389/fnins.2016.00563)
Supplement: Supplementary file 1 [file Image1.pdf]

*Supplementary Material*

# A bidirectional brain-machine interface featuring a neuromorphic hardware decoder

**F. Boi [1*+], T. Moraitis [2+], V. De Feo [1+], F. Diotalevi [4], C. Bartolozzi [3], G. Indiveri [2], A. Vato [1]**

[1]*Istituto Italiano di Tecnologia, Neural Computation Laboratory, Rovereto, 38068, Italy*

[2]*Institute of Neuroinformatics, University of Zurich and ETH Zurich, Zurich, Switzerland*

[3]*Istituto Italiano di Tecnologia, iCub Facility, Genova, 16163, Italy*

[4]*Istituto Italiano di Tecnologia, Robotics, Brain and Cognitive Sciences, Genova, 16163, Italy*

[+]*Equal contribution*

Correspondence*: Fabio Boi Istituto Italiano di Tecnologia, Neural Computation Laboratory, Rovereto, 38068, Italy, fabio.boi@iit.it

## 1 Supplementary Figures and Tables

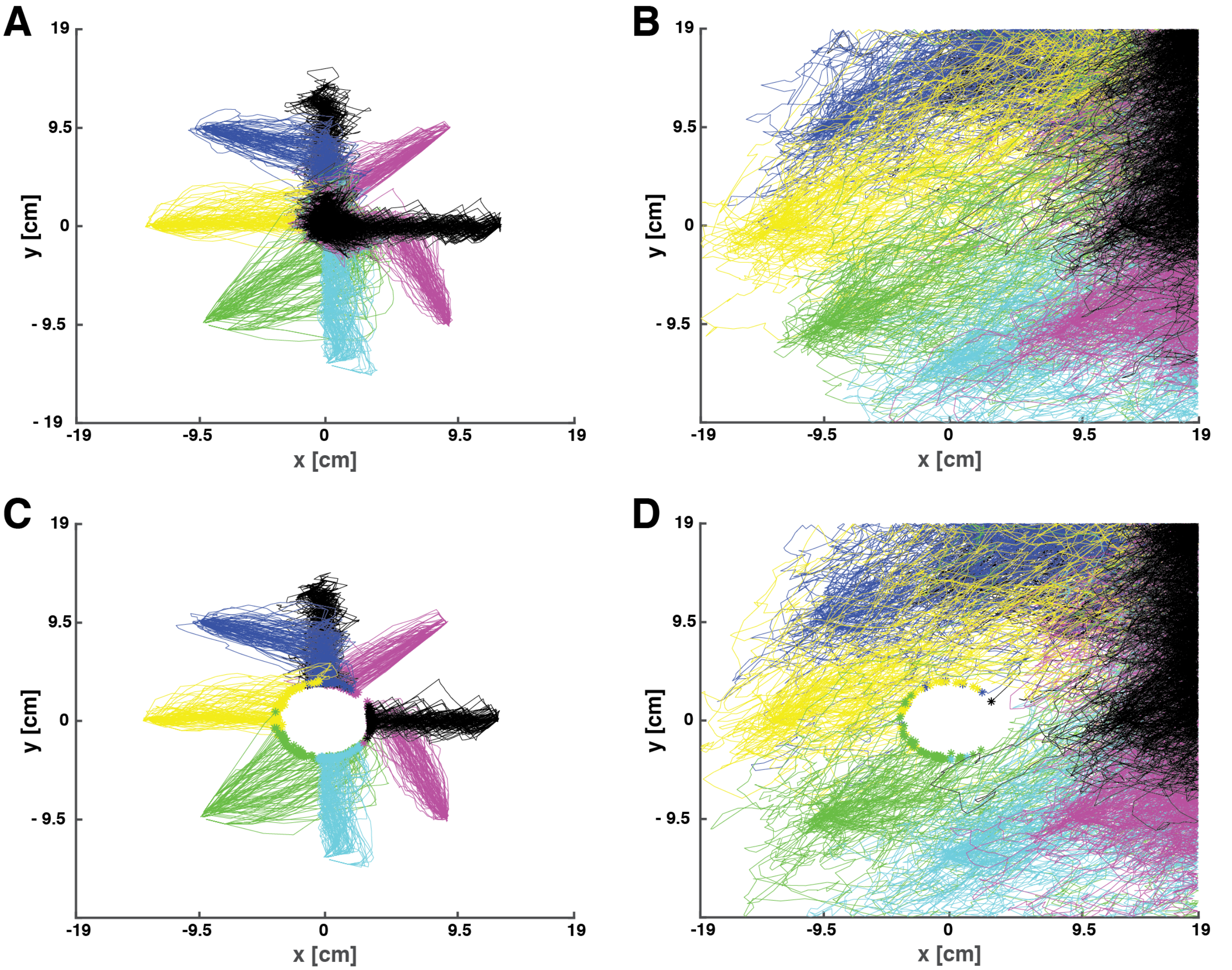


**Figure S1.** Experimental set-up testing. This figure shows the trajectories obtained by running the BMI 100 times from each starting point (i.e. 800 trials in total) and in the two conditions with encoder switched “ON” and “OFF”. (A) Trajectories obtained with the encoder “ON”. Each trial generates a trajectory of 100 decoding-encoding steps and the trajectories are represented with a different color for each different initial point. (B) Trajectories formed with the encoder “OFF”. (C) Trajectories obtained with the encoder “ON” and used to plot the figure 8A. The end point of each converging trajectories is designed with an “*” and represents the first point inside the target region. (D) Trajectories obtained by switching “OFF” the encoder by using the target-region stopping rule.
